# Digital Pathology: New Initiative in Pathology

**DOI:** 10.3390/biom12091314

**Published:** 2022-09-17

**Authors:** William C. Cho

**Affiliations:** Department of Clinical Oncology, Queen Elizabeth Hospital, Hong Kong, China; chocs@ha.org.hk or williamcscho@gmail.com

Digital pathology (DP) is an emerging field of pathology that manages information generated from digitized specimen slides. The information on the slides is converted into digital slides that can be viewed, managed, interpreted, analyzed, and shared in a digital environment. DP plays an emerging role in delivering anatomical pathology diagnostic images for electronic health records, telepathology, and further analysis on slides. In the practice of artificial intelligence (AI) and whole-slide imaging, DP is expanding its application to diagnosing and predicting prognosis and treatment response. Therefore, this Special Issue aims to collect the latest research and showcase the diverse applications of DP.

Bencze et al. [1] investigated a recently established AI-assisted digital image analysis platform, Pathronus, and compared it with five observers’ routine scoring of chromogenic immunohistochemistry (IHC)-stained slides. The accuracy of these methods was assessed by comparing statistical significance among groups to quantitative fluorescent IHC reference data on subsequent tissue sections. Ultimately, the authors found that AI-assisted software can identify cells of interest, distinguish organelles, protein-specific chromogenic labeling, and nuclear counterstaining, and thus provide a viable and accurate alternative to semi-quantitative scoring.

Microsatellite instability (MSI) is a useful biomarker for colorectal cancer when immunotherapy drugs are used [2]. Bustos et al. [3] reported an adversarial network-based multiple-bias-rejecting deep learning system for predicting MSI in colorectal cancer from tissue microarray. The system was trained and validated on 1788 patients from EPICOLON and HGUA. The authors claimed that this is the first work to incorporate multi-bias ablation techniques in the deep learning architecture of DP and the first to use tissue microarray for the task of MSI prediction. They found that the system combined a tissue-type classifier module to select regions of interest and an adversarial training-based multiple-bias rejection technique. The features learned from the bias ablation model were mostly discriminative for the MSI prediction task and had the smallest statistical mean dependence on bias.

Ki67 is an important biomarker with prognostic and potential predictive value in breast cancer [4]. Boyaci et al. [5] investigated the reproducibility among pathologists according to the Ki67 guideline of the International Ki67 in Breast Cancer Working Group (IKWG) and evaluated the prognostic potential of this platform in an independent cohort. In fact, the authors claimed that their study is the first independent validation of the IKWG guideline, with multiple observers. Four algorithms were independently built using the open-source digital image analysis platform (QuPath) according to detailed guidelines from the IKWG. Comparing each machine reading score with recurrence-free survival, they found similar hazard ratios. They demonstrated that good reproducibility among pathologists can be achieved using the IKWG automated Ki67 scoring guideline, achieving intraclass correlation coefficient values similar to those in the IKWG study. Furthermore, they demonstrated the prognostic potential of the automated IKWG scoring guideline in an independent breast cancer cohort.

Current methods for histological quantification are operator- and organ-specific. Courtoy et al. [6] developed a robust, minimally operator-dependent and tissue-transposable digital method for fibrosis quantification. The proposed method involves a new algorithm for a more specific and sensitive detection of picrosirius red-stained collagen fibers, a computer-aided segmentation of histological structures, and a new automatic morphological classification of fibers based on their compactness. The authors found that their new algorithm proved to be more accurate than classical filtering using the primary color components (red–green–blue) for picrosirius red detection. In conclusion, the team developed a powerful digital method for fibrosis analysis that allows accurate quantification, pattern recognition, and multi-organ comparisons to understand fibrosis dynamics.

On the other hand, Marti-Aguado et al. [7] presented a prospective, multicenter study including 156 patients with chronic liver disease. The aim of this study was to evaluate the relationship between DP analysis and the corresponding pathologists’ grading scores for the assessment of hepatic necroinflammatory activity. They performed whole-slide digital image analysis based on IHC color (CD45+) and morphological features to measure staining intensity areas (I-score) and clusters of staining intensities (C-score). Both I-score and C-score increased with inflammation grade and fibrosis stage, showing a good correlation with scoring by pathologists. The developed scores performed better than the other DP algorithms, reflecting the importance of a morphometric assessment [8,9]. The authors concluded that DP allows an automatic, quantitative, and morphometric assessment of hepatic necroinflammatory activity. It can serve as a potential aid to pathologists evaluating chronic liver disease biopsies in clinical practice.

AI and machine learning tools are increasingly being used to integrate clinical, histopathological, and genomic data [10]. Moran-Sanchez et al. [11] obtained metadata in this field from the Clarivate Analytics Web of Science database from 1990 to 2020. A total of 525 documents were assessed by document type, field of study, source title, organization, and country. The SciMAT and VOSviewer software packages were used to perform scientific mapping analysis. They found that the United States and China are the most productive countries. Current research focuses on the integration of digital image analysis and genomic sequencing in non-Hodgkin’s lymphoma, the prediction of chemotherapy response, and the validation of new prognostic models. The authors concluded that these findings can not only map future clinical and research pathways for the pathology sector, but also promote synergies and optimize funding allocations for public agencies and administrations.

In summary, DP enhances the workflow of telepathology in slide management, including scanning, viewing, networking, analysis, integration, and sharing. From this Special Issue, we can see the usefulness of computer algorithms in classifying tissue on digitized slides. However, validation of the digital microscopy workflow is important to ensure high diagnostic performance. As a result, the College of American Pathologists has issued a guideline with minimal requirements for validating whole-slide imaging systems for diagnostic purposes in human pathology [12].

DP has been approved by the United States Food and Drug Administration for primary diagnosis [13], and I envision more applications for DP in disease diagnosis and prognosis will follow. Together with other omics platforms, it is possible to integrate not only tissue-specific structures, but also discoveries of molecular biomarkers (such as mutated genes, tumor mutational burden, or transcriptional changes). Furthermore, in the process of the spatial analysis of tissue slides, multi-omics and multi-dimensional analyses of the tissue microenvironment will be more comprehensively developed towards precision medicine [14].
